# Challenges for Resuming Normal Life After Earthquake: A Qualitative Study on Rural Areas of Iran

**DOI:** 10.1371/currents.dis.b4e84b942500e2f8f260f3471b7ee815

**Published:** 2014-10-17

**Authors:** Fardin Alipour, Hamid Reza Khankeh, Hussain Fekrazad, Mohammad Kamali, Hassan Rafiey, Pooria Sarrami Foroushani, Kevin Rowell, Shokoufeh Ahmadi

**Affiliations:** Department of Social Work, University of Social Welfare and Rehabilitation Sciences, Tehran, Iran; Department of Health in Emergency and Disaster, University of Social Welfare and Rehabilitation Sciences, Tehran, Iran; Department of Clinical Science and education, Karolinska Institute, Stockholm, Sweden; Department of Social Work, University of Social Welfare and Rehabilitation Sciences, Tehran, Iran; Department of Social Work, University of Social Welfare and Rehabilitation Sciences, Tehran, Iran; The University of New South Wales, Sydney, Australia; Psychology & Counseling, University of Central Arkansas, Conway, Arkansas, USA; Department of Social Work, University of Social Welfare and Rehabilitation Sciences, Tehran, Iran

## Abstract

Background and objective: Growing evidence is indicating that some of disaster affected people face challenges to resume normal life several months after an earthquake. However, there is no sufficient in-depth understanding of complex process of resuming normal life after an earthquake in Iran, as one of the most disaster-prone countries in the world, and in rural areas as a particular setting. This study aimed to explore challenges of return to normalcy in rural earthquake-stricken areas of Iran. Methods: The study was conducted using qualitative content analysis method (Graneheim approach). Twenty people from the earthquake-stricken areas and seven qualified experts were selected via purposeful sampling .Data was collected through semi-structured interviews, focus group discussions, and field notes from August 2013 to January 2014. Data collection continued to the point of data saturation (no new information was provided by interviewees). Data saturation supported the sample size. Data analysis was based on qualitative content analysis principles. Results: “Social uncertainty and confusion” was the most prominent challenge of return to the normal life after earthquake, which was categorized into six concepts of social vulnerability, lack of comprehensive rehabilitation plan, incomplete reconstruction, ignorance of local social capital, waste of assets, and psychological problems. Conclusions: Findings showed that social uncertainty and confusion occurs as a result of negligence of some important social aspects in process of returning to the normal life. This issue, in turn, can greatly interrupt the normal developmental processes. Understanding the challenges of life recovery after disasters will help policy makers consider social rehabilitation as a key factor in facilitation of return to normal life process after earthquakes. Keywords: Disaster; earthquake; social rehabilitation; social uncertainty.

## Introduction

Disasters are of significant encounters for human societies worldwide and can have devastating social, medical, and public health consequences 1. Iran is highly vulnerable to different types of natural disasters such as earthquake, flood, and droughts. On average, 2000 to 3000 people are killed annually due to such incidences 2. The Rudbar-Manjil earthquake (1990), Bam earthquake (2003), Golestan flash floods (2000-2005), Lorestan earthquake (2006), Azarbaijan earthquake (2012), and Bushehr earthquake (2013) were of the most destructive disasters during last decades.

People’s life after natural events and disasters has not been studied sufficiently 3 ^,^ 4. Most of the studies in this field have only focused on short periods of disasters and long term processes were completely missed out. However, getting back to normal life after a disaster can be as significant as life in pre or during disaster stages. Although many experimental and theoretical works have been conducted on consequences of disasters, an in-depth understanding of rehabilitation and what exactly forms this process is still evolving 5. It is also suggested that the concept of rehabilitation cannot be decontextualized and investigated out of complicated economic, political, and social systems in which it happens^6^^,^ 7. Hence, to evaluate the challenges of rehabilitation process, it is necessary to have a multi-dimensional look rather than a one-dimensional perspective. Nevertheless, many of the studies conducted in this field have not taken a comprehensive look at this process, and have usually focused only on one aspect such as psychological interventions after disasters, stress disorders after disasters 8 ^-^ 11, physical injuries 12 ^,^ 13, the role of social capital in reconstruction 14, and the role of community participation in physical reconstruction 15. Consequently, the majority of rehabilitation plans are not comprehensive and there are limitations in adopted social approaches. They usually do not appropriately consider the variable range of long-term physical and social needs of societies after disaster occurrence 16.

Furthermore, research indicates that some people experience a disaster posttraumatic growth in terms of greater appreciation of life, deeper meaningfulness, closer relationships, and deeper religious faith; however, these studies do not describe the process of moving from disaster period to posttraumatic growth 17.

On the other hand, there is currently no comprehensive study on the concerns of life after disasters in Iran. Previous works have mainly focused on post-disaster mental health interventions 18, disaster relief problems and management inconsistencies 19 ^-^ 22, and healthcare challenges in disasters 23 ^-^ 25. However, most of these studies have not comprehensively evaluated the challenges of getting back to normal life after disasters, and more importantly, they have not used a qualitative approach to better understand the experiences and perceptions of disaster survivors. Thus, there is still a significant gap in this field. Clearly, this stage is a complicated, systematic, and interactive process which needs a more complete understanding of the long-term recovery experiences of people involved. The current study, therefore, investigated the challenges of returning to the normal life after earthquake in rural areas of Iran.

## Materials and Methods

**Study design: **A qualitative approach using content analysis (Graneheim approach) was considered appropriate for our study. In this method, required data was gathered directly from participants, without any pervious hypothesis. Produced knowledge was based on unique viewpoints of participants. Codes and categories were derived through an inductive process and then conceptually ordered properties and dimensions were developed 26.****

**Setting and participants**: The study was conducted in rural areas of Azarbaijan where there were two, somehow, massive quakes in 2012. According to their firsthand experience or expertise in earthquake and also their willingness to participate in the study and to gather in-depth and rich experiences, the participants were chosen using a purposive sampling method with maximum diversity. Sampling continued until data saturation was achieved. Of participants, 20 people experienced the earthquake firsthand and 7 people, including 3 social workers, 2 psychologists, and 2 local health workers, had scientific expertise in disaster recovery. Also, 2 focused group discussions were held to complete the data collection and understand the meaning of primary results. 11 local survivors attended the first group discussion session, 10 disaster recovery experts participated in the second session.

**Data Collection**: Semi-structured interviews, focused group discussions and field notes were used to collect data. Data collection was done by the main researcher who lived in earthquake-stricken area for about 18 months (August 2013 – January 2014). Before the interviews, by introducing himself and expressing the aim of the study, the researcher obtained the informed written and oral consent of the participants. The interviews lasted for 30 to 60 minutes and were tape recorded and transcribed verbatim; each interview began with a broad question about participants’ experiences of events they had observed. Probing was conducted according to reﬂection of each participant on life after disaster, such as perception of reconstruction process, services and their needs, facilitators and barriers of providing services, role of social networks, and organization of disaster recovery. The focus groups were run by the researcher and local assistants and attempts were made to facilitate discussions and consider all relevant sides of the issue.

**Data Analysis**: Qualitative content analysis was used to analyze the data. Systematic stages were followed and simultaneous analysis was undertaken: first, recorded interviews were transcribed verbatim. Then, before coding, the transcribed text was read several times for familiarization. Codes and categories were extracted by an inductive process via open coding (through line by line reading of the text and devoting relevant codes to it). Then, categories emerged by constant comparison. Peer check and constant comparison were used to reach a consensus in coding. In fact, data analysis was performed simultaneously and continually with the data collection. After completion of coding and assuring accuracy of coding, concepts were identiﬁed.

**Trustworthiness:** we used the strategies recommended by Lincoln and Guba for trustworthiness. According to this recommendation, four criteria of creditability, dependency, conformability, and transferability are necessary for trustworthiness. To increase data creditability, the researcher engaged with data and the environment for 18 months while constantly making observations and compiling field notes. Dependency of data was assessed by peer check strategies. Peer check was performed on a monthly basis so that the research team had a thorough discussion about emerged data. The background and personal interest of researcher on the subject and maintaining documents of study were used for conformability of data. The context of the interviews, codes, and the extracted categories were reviewed by the research team and other professional colleagues in the field of qualitative research. Using sampling with a maximum variation, the researchers were able to collect a wide variety of different comments, observations, and interpretations.

**Ethical Considerations**: Informed consent was obtained through explaining the aim and process of the study orally and in written. The study was approved by the ethical committee of University of Social Welfare and Rehabilitation Science (USWR) in Tehran, Iran. Information was kept conﬁdential and Participants had the right to withdraw at each stage of the study. .

## Results

Of 27 participants of the study, including survivors of the earthquake-stricken areas and the qualified people with relevant scientific expertise, the mean age was 41 years. 21 of participants were men, three were single, and the literacy level ranged from illiterate to postgraduate qualifications. Quotations mentioned in this article are all translated from Persian into English by the researcher (FA). Accuracy of translations was independently checked by another author (PSF).

Findings indicated that “social uncertainty and confusion” was the main concern of people after disaster, a matter which was directly and indirectly hidden in most of study codes. People reported that they lived with significant uncertainty about the future and that they experienced a significant decline in perceived ability to recover and in motivation. One of participants explained the situation as follows:

“…*before the earthquake we were all busy with our everyday life. We knew what to do, where to go, what we were looking for …but after this long period from the earthquake, we just spend days and nights and think about all of the our troubles. We feel that we do not know what we are doing, where we are going, and finally what will happen to us. We are tied up to these troubles and unfinished jobs. Our Houses are ruined and agriculture and husbandry are destroyed. Finally, we will be forced to leave this place (Participant No. 8; resident, man, 4o years old).”*

This study also identified six other main concepts related to participants’ main concerns: social vulnerability, lack of comprehensive rehabilitation plan, ignorance of local social capital, waste of assets, and psychological problems. These main categories and their subcategories are explained below:

**1. Social Vulnerability**

Findings showed that disasters are associated with everyday life of people. The severity of damages and their consequences depend on various factors such as socioeconomic vulnerability of people. Experiences and perceptions of the participants indicated that natural events are not the only obstacles impeding their return to normal life and different direct and indirect factors affect the speed and quality of this process. Three emerged subcategories about social vulnerabilities were as follows: 1) inefficiency of local non-governmental****social institutions, 2) lack of public awareness, and 3) social problems before the earthquake.

**1.1 Inefficiency of local non-governmental social institutions**

According to the results, inefficiency of non-governmental social institutions had left the government alone with the responsibility of restoration, and caused various problems in returning to normal life. Inefficiency of institutions such as councils and associations caused challenges for people in providing assistance and communicating with governmental institutions. From the viewpoint of participants, intermediary institutions could facilitate the rescue works, make them more goal-oriented, and prevent from lots of financial waste. It is noteworthy that competency of intermediary institutions is also an important factor. If people do not trust them or if they are incompetent, then their effectiveness is lost. One of the participants explained the situation as:

“…in some areas we don’t have any intermediary institutions to play an active role in post disaster recovery and in other areas there are problems in relation between people and these institutions. From my viewpoint, since the rescue work should be mainly done by these people and their systems, their competency is important. Their incompetency was one of the problems in some areas, which made rescue work and goods distribution more difficult …” (Participant No. 18; resident, woman, 49 years old).

**1.2 Lack of public awareness** **

Lack of public awareness was another subcategory which caused problems for people at disaster time and even during the process of getting back to normal life. Most of the participants talked about lack of in advance knowledge and skills to respond to and recover from a disaster; they also believed that the current public education was not effective enough and made them unable to play any active role. A participant noted the issue as:

“…it is noteworthy that shortly before the earthquake, they held earthquake training here and we participated. Of course, it was a fake training and was not held seriously. Most of the people even were not aware of it and those who participated were only observers. They burned a car’s tire and then extinguished it while laughing and joking … and called it the readiness training and exercise for natural disasters”(Participant No. 23; community health worker, man, 4o years old).

**1.3 Social problems before the earthquake**

Pre-earthquake social problems significantly increase vulnerability and hinder people’s return to normal life in a timely fashion. Most of participants believed that factors such as poverty, inequality, and unemployment were factors which increased their social vulnerability and created problems in returning back to normal life in an efficient manner:

“…the problems and challenges that we had before the earthquake are still with us, making our lives more difficult. The condition for people who did not have a good financial situation before the earthquake is worse than others and they still couldn’t get back to their normal life.”(Participant No. 4; resident, man, 65 years old).

Participants believed that people who had physical, social and, personal vulnerabilities suffered the most losses:

“…people who did not have a good financial situation, or had shouldered more losses, or have lost one of their close relatives are currently having more difficulties and should be protected…” (Participant No. 21; rescuer, man, 38 years old).

**2. Lack of comprehensive rehabilitation plan**

Findings showed that there were times of progress and yet times of frustration in the process of helping people to get back to normal life. In some areas, the management was very effective, yet in others it was very poor having no leadership or even being totally ignored. Participants believed that it is necessary to determine responsibility of the teams and consider appropriate measures before, during, and after disasters. Regarding this issue, findings revealed four subcategories of: 1) lack of comprehensive data and information, 2) negligence of vulnerable groups, 3) Concentration on reconstruction and overlooking of rehabilitation, and 4) improper distribution of resources.

**2.1 lack of comprehensive information**

One of the obstacles impeding the process of rehabilitation was the lack of comprehensive information about the demographics of the people and residents of each district. This lack of this information was significantly noted in views of a rescuer:

“…unfortunately, since the responsible organizations lacked a detailed statistical system the rescue problems were intensified, and unfortunately some of the people who are residing here in the shelters are not the permanent residents of the village and only used to live here seasonally; we do not have any detailed statistical data about them. For example, a village had 80 households and we only had 45 tents, which caused a chaos among the people...” (Participant No. 27; rescuer, man, 42 years old).

**2.2 Negligence of vulnerable groups**

Neglecting vulnerable groups was another subcategory extracted in the study. Findings showed that certain groups had not been considered in the distribution of goods, nor had their specific requirements been considered. Women, children, the elderly, and people with disabilities were among the groups that, according to the participants, were greatly neglected. This significantly led to intensification of social confusion and delayed rehabilitation. Rescuers had firsthand views of negligence of these subgroups. One rescuer explained the situation as:

“…we were in a village to distribute women basic hygiene items. However, women felt embarrassed to receive them from us because we were men. Finally, at our return, many of them thanked us and stated that these items were more important for them than rice, food and etc…” (Participant No. 24; recovery service provider, man, 26 years).

One of the female participants said:

“…most of the times, the relief goods were distributed in a way that we couldn’t get them. Because men were stronger and could get more goods, but women, the elderly and the others who did not have such power couldn’t get them. Even sometimes we were embarrassed…” (Participant No. 5; resident, woman, 42 years).

**2.3 Improper distributions of resources**

Another extracted subcategory was the improper distribution of resources. In many cases, because of improper management much of resources were wasted thereby leading to more difficulties among the survivors:

“Sometimes they provide us some food, but there is no regulation in distribution. Our relatives also bring us some food from the city. Sometimes benefactors also provide us something. But the most outstanding defect is lack of an organized program. Packs of water or canned fishes are stacked in one place and wasted, while in some other places people are in a great need of these items…”(Participant No. 24; recovery service provider, man, 26 years).

**2.4 Concentration on reconstruction and overlooking of rehabilitation**

Another subcategory extracted was mere concentration on reconstruction and overlooking of rehabilitation. People, authorities and recovery services focused only on reconstruction, with little attention to rehabilitation needs of the people. This led to intensification of social uncertainty and confusion in the earthquake-stricken areas, as explained by one of the participants:

“…the government has done its best to reconstruct the houses and this has not been completed yet and most of the houses are incomplete, but unfortunately no attention has been primarily paid to agriculture, husbandry, employment, and mental, and social issues ... ”(Participant No. 19; resident, man, 66 years).

The people of earthquake-stricken areas also concentrated on reconstruction and did not attend to other aspects of recovery. In this regard one of the participants said:

“…but here people are concentrated on the houses. If the houses are completed, people can get back to their normal life. We are only busy with reconstructing our homes and working. Therefore, we cannot do anything else. We urge the government only to take the reconstruction seriously. If they complete our houses, we will be relaxed…”

** **3. Incomplete reconstruction**

Participants believed that getting back to normal life depends on the completion of reconstruction and therefore they were worried about the delay of this process. Most of the participants emphasized the importance and priority of physical reconstruction and believed that their return to normal life significantly depended on completion of physical reconstruction.

Our analysis revealed two subcategories regarding incomplete reconstruction: 1) failure of timely reconstruction, and 2) Construction without considering the culture

**3. 1 Failure of timely reconstruction**

Failure of timely reconstruction due to different reasons was one of the main factors contributing to confusion in returning to normal life. Based on participants’ viewpoint, one of the main reasons for this problem was the failure to control and supervise reconstruction and also break of contractors who had left the projects incomplete during the cold season so that people could not get back to their normal life:

“…we are currently involved in reconstruction of our houses and there is nothing else and we cannot have proper food. The only thing I have to think about now is finishing my house to stay safe and sound against cold. If I get stuck with construction and don’t finish it soon, I will not be able to start working on agriculture and husbandry next year” (Participant No. 15; resident, man, 50 years old).

From the people`s point of view, completion of reconstruction would be a necessary relief from their current situation and they believe that it would be a way to gradually forget the bitter memories of earthquake. However, a mass of rubble on the road side of many villages was a reminded of earthquake which left people with feelings of depression:

“…every time I pass this road I see the rubble on the road side and the bitter memories of the quake come to my mind again…” (Participant No. 6; resident, man, 18 years).

**3. 2 Reconstruction without considering the culture**

The second subcategory, the inability to consider local cultural needs, led to problems in recovery. Negligence of special requirements in rural houses was one of the main aspects of reconstruction that led to dissatisfaction and uncertainty in people’s lives. In other words, architecture and interior design of the reconstructed houses did not meet the needs of people in earthquake-stricken areas. They were just shelters and in many cases had no bedrooms, workshop, or barn. The reconstruction itself was very satisfying for people, but lack of attention to cultural aspect of reconstruction was one of the main concerns. A participant argued that they have especial problems with houses:

“We need large rural houses. We usually have many guests and these sizes are insufficient. They have built us some civic houses. These houses might be suitable for people who live in cities, but are not enough for us. The houses have not enough bedrooms and no place for carpet weaving. Husbandry and agriculture have been also neglected. As you enter the village these houses look beautiful, but if you put yourself in my place, you will see these houses are like a cage...” (Participant No. 14; resident, man, 34 years old).

** ****4.**** **Ignorance of local social capital**

An important aspect in disaster recovery and return to normalcy is that survivors need to be active participants in the process. Self-efficacy is an important concept in psychological health, but when people are not included in their recovery; their sense of self-efficacy can be substantially undermined. Regarding the issue, following subcategories were extracted: 1) a top-down paternalistic approach, 2) undermining of trust, and 3) undermining of social networks and generation of self-centering.

**4.1 A top-down paternalistic approach**

Findings support the idea that even regarding the minor issues in recovery, people were not asked for their ideas and input, nor were they asked to participate in reconstruction and other aspects of rebuilding. Most of the plans have been performed following a top-down paternalistic approach. It caused people to feel dissatisfied and not to have the sense of ownership and belonging regardless of several governmental efforts that were made for them. In this regard, one of the participants said:

“…only during the first days after the earthquake people sometimes helped each other, and then we just received relief goods and people played no role. We were all waiting for the Red Crescent and other institutions to come and help us…” (Participant No. 9; resident, man, 60 years old).

Another participant explained the situation as bellow:

“…the situation is not like before at all. They only built us some 60 m^2^ houses and now they want us to go and live in them. We have no choice but to tolerate this situation, but have no sense of ownership to these houses. These houses cost us a lot and we were forced to sell everything (to buy the houses)…” (Participant No. 11; resident, woman, 58 years old).

**4.2 undermining of trust**

Another extracted subcategory was undermining of trust. Before and after the disaster, a kind of distrust was built up between the earthquake-stricken people and the government, and also between benefactors and the government for various reasons. According the participants, these conditions paved the way for confusion and social uncertainty:

“…if the government did nothing and just gave us the benefactors’ reliefs, we had no problem and everything was good. The government seized all the money and relief goods of benefactors’ and instead gave us loans. Instead of receiving an aid from the government, I am in debt of a 25 million Tomans loan to the government” (Participant No. 15; resident, man, 50 years old).

In addition, there was another kind of distrust between benefactors and the government that led to special problems. In fact, the donations were distributed by benefactors and these distributions were not targeted and organized. A participant described the situation as:

“…We must accept the fact that many people do not trust government agencies. Why not to trust is another matter. In fact, the government agencies should figure out why people intend to directly aid earthquake-stricken areas. It is necessary to study why they do not deliver their aids to the governmental agencies…” (Participant No. 21; psychologist, man, 38 years old).

One of the community disaster recovery staff described the situation as:

*“* *Unfortunately, benefactors* *treat quite emotionally so that they directly deliver their aids to the villages or those who pretend to be needy villagers. In Iran, in the early stage of disaster recovery management, aids of donors and responsible organizations are like a flood, they suddenly come and go. If it is not effectively managed, it can lead to waste of aids“(Participant No. 20; resident, man, 27 years old).*

**4.3 undermining social networks and generation of self-centering**

The third subcategory of social capital neglect was the undermining of social networks and creation of self-centering. According to some participants, this was a significant obstacle in their ability to gain social effectiveness and a successful return to normal life:

“…during the first days after the earthquake people helped each other, especially they rescued many victims by helping each other. Unfortunately, as time passed, especially during the reconstruction of houses and receiving of relief goods, people just thought about themselves and everyone tried to get more goods…”(Participant No. 7; resident, man, 41 years old).

**5. Waste of Assets**

Waste of people`s critical assets was another factor that impeded recovery. Some people were forced to sell their cattle at low prices after the earthquake because they did not have adequate barns and shelters necessary to take care of their livestock. These assets later were very important for getting back to normal life. Yet, most people were not able to make the loss up after some months because they could not afford the higher prices for cattle:

One of the participants explained the situation as:

“…since all infrastructures were destroyed and there were no facilities, people were forced to sell their cattle. May people came here and bought our assets. We waited a month for a barn and when they didn’t build it, we were forced to sell our cattle. Dealers took advantage of our situation…” (Participant No. 2; resident, man, 28 years old).

Loss of properties and assets caused people to think the earthquake has seized everything they had, so they lose their hope to get back to the normal life. Shortly after earthquake, conditions had changed in a way that people thought they cannot revive their lost assets. For example one participant said:

*“…of course we want to get back to our home, but how? We do not have anything. I had 10 cows and was forced to sell them in those days, and now having those cattle is like a dream, which cannot be achieved. These small houses and high prices do not let us buy even two cows …” (Participant No. 14; resident, man, 34 years old)* *.*

** **6. Psychological Problems**

Most of participants were challenging with significant psychological problems as one of the consequences of earthquake. Even after several months, different groups of people suffered from various mental problems such as depression, anxiety, and fear. In many cases, fear and anxiety led to sleep disorder among children. One of the participants described it as:**

“The earthquake mentally affected us, especially the children. Now children are afraid of every little sound. They are very sensitive. We cannot have a good sleep, every moment we are afraid of another earthquake. There were numerous aftershocks; there was one some nights ago …” (Participant No. 18; resident, woman, 49 years).

18 about months after the earthquake, many participants talked of lack of desire to get back to the normal life, feeling sad and …:

“…we are not like before. We have lost our motivation and most of the people are not happy and are mostly sad. If you look at our situation from distance, you cannot recognize the sheer reality of our lives. It takes a life time to get back to previous point” (Participant No. 11; resident, woman, 58 years old).

## Discussion

The current study, which was the first of its kind using a qualitative and comprehensive approach, evaluated the challenges of people in return to a normal life after a significant earthquake in rural areas of Iran. By analyzing viewpoints of earthquake victims and disaster experts dealing with rehabilitation interventions, the study provided a much needed perspective of the most important problems of post-disaster recovery process. The key element explored in this study was “social uncertainty and confusion”. Furthermore, the findings identified six inhibiting factors or barriers to successful recovery, namely, lack of a comprehensive rehabilitation plan, social vulnerability, ignorance of local social capital, incomplete reconstruction, waste of assets, and psychological problems. These issues greatly prolonged the rehabilitation process thereby causing people to suffer from social uncertainty and confusion.

One of the main obstacles on the way back to normal life after earthquake was the lack of a comprehensive rehabilitation plan. Participants indicated that the recovery process started with an emotionally-laden management approach with a lot of energy, but gradually the momentum declined until it reached a level of no further activity thus leaving people bewildered, confused, and forgotten. Another important issue was the presence of unresolved social problems before the earthquake which led to intensification of social uncertainty and confusion in the aftermath of the disaster. This finding was consistent with those of other similar studies 27 ^-^ 30.

Social capital can be conceptualized as social connections between people occupying homophiles networks, across heterogeneous networks and organizations, and with those of higher status and power 31. Accordingly, our findings also indicated that failure to fully engage community in recovery strategies and efforts was another reasons for social uncertainty and confusion. As relief agencies did not encourage participation, people felt a reduced sense of belonging, greater dissatisfaction, and more dependency on governmental assistance. Importance of public participation in post-disaster rehabilitation has been also emphasized in other studies 14 ^,^ 15.

Lack of occupational infrastructures was another issue in return to normal life. Absence of a systematic program in this regard and lack of income led to financial distress among people. Financial distress was also occurred as a result of forced sale of assets, properties, and livestock at low prices immediately after the earthquake and the subsequent failure to recover them due to high pricing in the recovery period. Pyles, (2007) have similarly emphasized issues surrounding restoration of jobs and businesses after disasters 32.

Findings indicated that rebuilding of homes and infrastructures were of main preconditions of reconstruction in other spheres of rehabilitation. Delays in process of rebuilding led to delays in other social, psychological, and economic aspects. Speed and quality of reconstruction were specifically identified as the core problems. These findings were similar to other disaster related studies 33 ^-^ 35. This was very interesting that all governmental and lay people focused only on reconstruction and did not care about rehabilitation.

Gender is an important variable among social factors; disaster research has indicated that women generally are more vulnerable to disasters 36. The study also found problems and limitations in providing services, specifically for women and other vulnerable groups. Due to lack of awareness or insensitivity to needs of these groups, they were greatly underserved which further compounded their social uncertainty and confusion. Zahran and colleagues have concluded that domestic violence would increase after disasters 37. Such an issue was not mentioned by the present study`s participants. Authors think that such issues need to be studied in future and with relevant methodologies.

Finally, as typically seen in all disasters, people experienced various kinds of psychological and emotional disturbances. Depression, anxiety, and fear of future earthquakes were the most common problems indicated 17; however, in some groups, especially children, sleep disturbance was a significant symptom.

## Conclusion

According to the results of the current study, an effective rehabilitation plan needs a comprehensive management system that first of all should be approved and understood by people, intermediary and governmental institutions, and other beneficiaries. In addition, an effective rehabilitation plan also needs a common understanding, considering the complicated and multi-dimensional, dynamic and long term nature of getting back to the normal life with maximum rate of people’s participation. Rehabilitation is prior to reconstruction, but still most of the people and disaster management affiliated organizations in Iran believe that the rehabilitation is a set of governmental interventions aiming merely at reconstruction. According to the results of the current study, this approach has to be reformed and considered in the further policies of rehabilitation as a social and developmental process. Rehabilitation should be considered as a comprehensive process to support affected communities with their maximum representation and aiming people to achieve the highest degree of independency and sufficiency.

Hence, policy-makers are recommended to change their viewpoints about rehabilitation from a linear and outcome-oriented approach to a continuous, prolonged, and comprehensive process. It is also necessary for policy makers to consider psychosocial interventions issues raised in the current study as a main part of disaster recovery plans, and especially they must change their lens and see psychosocial interventions as a main part of the disaster recovery programs not as a luxury and unnecessary intervention.

This study is one of the few studies on recovery and rehabilitation process that has employed the social approach. The biggest strength of the present study was that the main researcher, though facing many challenges, lived in the affected area and observed closely the process of community recovery for almost 18 months. However, data were collected from a limited sample of individuals using purposeful sampling, as dictated by the employed qualitative method. Therefore, the ﬁndings cannot be generalized to other locations. The present study marks a starting point for clarifying and describing the process of resuming normal life after earthquake. Further study on this process is needed. Studies including the perspectives of more affected people, policy-makers, and local authorities could yield increased understanding of this process. There is a need for further investigation of the process of life after disaster using Grounded Theory approach to develop strategies for improving disaster recovery systems.
