# Correction: Challenges for Resuming Normal Life After Earthquake: A Qualitative Study on Rural Areas of Iran

**DOI:** 10.1371/currents.dis.7118c63570e15df3cc033090ea4e0120

**Published:** 2015-03-09

**Authors:** PLOS Currents

## Correction

There are errors in the author affiliations. The affiliations should appear as shown here:

Fardin Alipour^1^, Hamid Rez Khankeh^2^, Hussain Fekrazad^1*^, Mohammad Kamali^5^, Hassan Rafiey^1^, Pooria Sarrami Foroushani^3^, Kevin Rowell^4^, Shokoufeh Ahmadi^6^

1 Department of Social Work, University of Social Welfare and Rehabilitation Sciences, Tehran, Iran

2 Department of Health in Emergency and Disaster and Nursing, University of Social Welfare and Rehabilitation Sciences, Tehran, Iran. Department of Clinical Science and Education, Karoliska Institutet, Stockholm, Sweden

3 University of New South Wales, Sydney, Australia

4 Department of Psychology and Counseling, University of Central Arkansas, Conway, Arkansas, USA

5 Department of Rehabilitation Management, Medical University of Iran, Tehran, Iran

6 Department of Health in Emergency and Disaster, University of Social Welfare and Rehabilitation Sciences, Tehran, Iran

*hamid.khankeh@ki.se

## Reference

Alipour F, Khankeh HR, Fekrazad H, Kamali M, Rafiey H, Sarrami Foroushani P, Rowell K, Ahmadi S. Challenges for Resuming Normal Life After Earthquake: A Qualitative Study on Rural Areas of Iran. PLOS Currents Disasters. 2014 Oct 17. Edition 1. doi: 10.1371/currents.dis.b4e84b942500e2f8f260f3471b7ee815.

View Article

