# Correction: Correction: Challenges for Resuming Normal Life After Earthquake: A Qualitative Study on Rural Areas of Iran

**DOI:** 10.1371/currents.dis.7c5047fd650abcb2339d1af7782a3066

**Published:** 2015-03-27

**Authors:** PLOS Currents

## Correction

Both the spelling of the corresponding author's name, as well as the designation are incorrect in the original correction. The corresponding author of this article is Hamid Reza Khankeh.
